# Evaluation of Pain and Oral Health-Related Quality of Life Associated With Fixed Orthodontic Treatment in Adults: A Systematic Review and Meta-Analysis

**DOI:** 10.7759/cureus.105336

**Published:** 2026-03-16

**Authors:** Omar A Rasol, Mohammad Y. Hajeer, Ahmad S. Burhan, Huda Abutayyem, Samer T. Jaber, Alaa Oudah Ali Almusawi

**Affiliations:** 1 Department of Orthodontics, Faculty of Dentistry, University of Damascus, Damascus, SYR; 2 Department of Clinical Sciences, Center of Medical and Bio-Allied Health Sciences Research, College of Dentistry, Ajman University, Ajman, ARE; 3 Department of Orthodontics, Faculty of Dentistry, Al-Wataniya Private University, Hama, SYR; 4 Department of Orthodontics, Faculty of Dentistry, University of Al-Knooz, Basrah, IRQ

**Keywords:** discomfort, fixed appliances, fixed orthodontic treatment, ohip questionnaire, oral-health-related quality of life, pain, quality of life

## Abstract

Fixed orthodontic treatment is commonly associated with pain and discomfort, particularly during the initial stages, which may influence patient adherence and satisfaction; however, a comprehensive synthesis of evidence, specifically focused on the adult population, remains limited. This systematic review aimed to evaluate pain and oral health-related quality of life (OHRQoL) in adult patients undergoing fixed orthodontic treatment.

Electronic searches were conducted up to August 2025 across multiple databases, including PubMed®, Web of Science™, Scopus®, Google™ Scholar, PsycINFO®, EMBASE®, and the Cochrane Library, with no restrictions on publication date or language. Observational studies, randomized controlled trials (RCTs), and controlled clinical trials (CCTs) involving patients aged 18 years or older, treated with conventional fixed appliances, were included. Two reviewers independently screened studies, extracted data, and assessed risk of bias using the RoB-2 and ROBINS-I tools. Meta-analysis was performed using RevMan 5.4 (The Cochrane Collaboration, The Nordic Cochrane Center, Copenhagen, Denmark), and the certainty of evidence was evaluated using the GRADE (Grading of Recommendations Assessment, Development and Evaluation) approach.

A total of 14 studies involving 1,022 adult patients were included, comprising seven RCTs, three CCTs, three cohort studies, and one cross-sectional study. The mean age of participants ranged from 18.87 to 38 years, with a female predominance of approximately 61%. Pain intensity peaked at 24 hours post-activation, with Visual Analog Scale scores ranging widely from 19.5 to 56.7. Meta-analysis of two studies revealed a statistically and clinically significant reduction in pain from day 1 to week 1 (mean difference = 20.83, 95% confidence interval: 11.70 to 29.97, p < 0.00001, I² = 67%), with pain continuing to decline over subsequent weeks. OHRQoL, primarily measured using the Oral Health Impact Profile-14, showed initial impairment, followed by significant improvement over time. However, the overall certainty of evidence for both outcomes was rated as very low to low.

In conclusion, fixed orthodontic treatment in adult patients is consistently associated with moderate-to-high pain levels that peak around 24 hours and decrease significantly within the first week, while OHRQoL follows a parallel pattern of initial decline, followed by measurable improvement within the first month. Clinicians may use these findings to counsel adult patients and set realistic expectations regarding the transient nature of initial discomfort. Given the very low to low certainty of evidence, there is an urgent need for more well-designed longitudinal studies, with standardized outcome assessment time points and reporting metrics, to strengthen the evidence base.

## Introduction and background

Fixed orthodontic treatment is a widely employed technique for correcting dental malocclusions and achieving optimal occlusal relationships [1]. Brackets, arch wires, and bands are the key components of a fixed appliance, which are attached to the teeth to exert controlled forces to guide tooth movement, providing precise control over dental positioning [1,2]. Although newer forms of fixed appliances, such as ceramic braces and self-ligating brackets (SLBs), as well as aesthetic alternatives like clear aligners and lingual fixed appliances (LFAs), have emerged, the conventional fixed metallic orthodontic appliance remains the gold standard in orthodontic treatment [2].

Pain, as defined by the International Association for the Study of Pain, is “a negative sensory and emotional reaction linked to actual or possible injury to body tissue” [3]. Pain and discomfort are common symptoms of orthodontic treatment. This may discourage patients from initiating treatment; it may also affect their cooperation and adherence to the treatment plan, and the orthodontist’s instructions, all of which may negatively impact treatment satisfaction [4,5]. In orthodontics, pain is caused by the inflammatory response within the periodontal ligament following the application of force, and may vary in intensity and duration across treatment phases [6]. The assessment of pain intensity associated with orthodontic treatment has become a central focus in current studies [7-9]. The most practical and widely accepted approach involves patient self-reporting, using standardized scales such as the Visual Analog Scale (VAS), Numerical Rating Scale (NRS), and Verbal Rating Scale (VRS) [10].

Oral health-related quality of life (OHRQoL) is a comprehensive concept that describes how oral health influences an individual’s physical comfort, emotional state, and social functioning. It includes factors such as pain, functional limitations, emotional distress, and social interactions resulting from oral conditions or treatment [11]. In orthodontics, OHRQoL has become a common outcome measure, as malocclusions and their correction can significantly influence patients’ self-esteem, daily functioning, and satisfaction with treatment [7,12-14].

Several systematic reviews have been conducted on pain and quality of life associated with orthodontic treatment [2,5,15-17]. The majority of systematic reviews have either failed to isolate adult patients as a distinct population or centered their comparisons on conventional appliances versus clear aligners [2,16,17]. The review by Inauen et al. addressed the topic of the pain profile in patients undergoing leveling and alignment by fixed orthodontic appliances; however, the authors included studies involving patients of mixed age groups [5]. Meanwhile, the review by Cheng et al. has focused on the effect of analgesics in controlling pain during orthodontic treatment, with the use of non-steroidal anti-inflammatory drugs (NSAIDs) being a key inclusion criterion for the selected studies [15]. The recent review by Johal et al. compared labial fixed appliances, palatal fixed appliances, and clear aligners in terms of pain, quality of life, and side effects in adult patients [18]. While their work provides a valuable comparison across multiple orthodontic modalities, a dedicated synthesis focusing exclusively on conventional fixed appliances in the adult population remains warranted. This leaves a significant gap in understanding how treatment with the conventional fixed orthodontic appliance affects adult patients in terms of pain experience and its impact on OHRQoL, as the majority of reviews have either failed to isolate adult patients as a distinct population or have focused on comparisons between several modalities. Adult patients differ physiologically and psychosocially from younger patients, with potentially heightened sensitivity to pain, and differing expectations regarding treatment outcomes [19].

Moreover, current reviews have not examined newer or less conventional alternatives, such as lingual fixed appliances or treatments employing acceleration techniques (physical or surgical), in comparison to conventional fixed appliances. Therefore, the current work aims to answer the following review question: what is the level of pain associated with fixed orthodontic treatment, and how does it affect the quality of life in adult patients?

## Review

Materials and methods

Preliminary Search and Protocol Registration

A pilot PubMed search was conducted before drafting the final protocol to verify that no comparable studies were available and to identify relevant literature. The protocol was recorded in PROSPERO during the initial stages of this study (CRD420251231931). This systematic review was carried out in accordance with the Preferred Reporting Items for Systematic Reviews and Meta-Analyses (PRISMA) guidelines [20,21] and the checklist and methodology recommendations provided in the Cochrane Handbook for Systematic Reviews of Interventions [22].

Eligibility Criteria

This systematic review explicitly defined its inclusion and exclusion criteria. The PICOS framework (population, intervention, comparison, outcomes, and study design) was used to define individuals, interventions, comparisons, outcomes, and study design. The target population consisted of adult patients aged 18 years or older who had undergone treatment with a conventional fixed metallic appliance. Regarding comparisons, no direct comparison was targeted in this review. However, studies that compare conventional fixed appliances to other orthodontic appliances were included if they reported relevant outcomes. The primary outcome of this review was patient-reported pain at the following time points: one day, one week, two weeks, one month, and three months. The effect on patients’ OHRQoL, measured using different scales at the same previous sequence of time points, was the secondary outcome. Observational studies (cross-sectional and cohort) were primarily targeted. Randomized controlled trials (RCTs) and controlled clinical trials (CCTs) were also included if they provided relevant outcome data. The following studies were excluded: those that did not specify adults as participants and included patients of all ages; those involving pain‑relief medications; studies with fewer than 10 participants in the experimental group; studies lacking sample reporting; case reports; case series; opinion pieces; reviews; and technique-description articles.

Information Sources

The electronic search keywords are presented in Table 1, while the full search strategy is detailed in Appendix 1. The initial search was conducted in August 2025 by two reviewers (OAR and MYH) without time restrictions, using PubMed®, Web of Science™, Scopus®, Google™ Scholar, PsycINFO®, EMBASE®, and the Cochrane Library. In addition, the reviewers manually examined the reference lists of all included studies.

**Table 1:** Keywords used in the electronic search

Aspects of the search strategy	Keywords
Type of malocclusion	Permanent dentition, permanent occlusion, malocclusion, class I malocclusion, class II malocclusion, class III malocclusion, dental malocclusion, crowding, spacing, overjet, overbite, cross bite, adults, adult patients.
Type of orthodontic tooth movement or orthodontic appliance	Leveling, alignment, extraction, retraction, canine retraction, anterior segment retraction, intrusion, extrusion, expansion, distalization, fixed orthodontic treatment, fixed metallic appliance, conventional metallic appliance, fixed appliance, clear aligners, Invisalign, self-ligating braces, lingual orthodontic treatment, lingual orthodontic appliance, lingual appliance, removable, removable appliance.
Outcomes	Pain, swelling, tension, pressure, soreness, pain perception, pain intensity, pain assessment, discomfort, pressure sensation, pain threshold, pain duration, VAS, visual analog scale, numerical rating scale, NRS, Likert, self-reported pain, oral health-related quality of life, OHRQoL, quality of life, patient-reported outcomes, oral health impact profile, OHIP, OHIP-14, psychosocial impact, functional limitations, daily life interference, self-esteem, patient experience.

Search Strategy and Study Selection

The study selection process went through two phases. Initially, two reviewers (OAR and MYH) independently screened the titles and abstracts of the articles retrieved from electronic databases, followed by a full-text evaluation of potentially eligible studies. Articles that failed to meet the inclusion criteria were excluded. In case of disagreement, the two reviewers consulted the third author (ASB) in order to reach a solution.

Data Collection Process

Two reviewers (OAR and MYH) extracted data from the included studies and compiled it into tables. The information gathered comprised general details (author and year of publication), study design, mean age, sample size, intervention types, follow-up period, and reported outcomes. Any disagreements between the reviewers were settled through discussion and, when necessary, consultation with the third author (ASB).

Risk of Bias Assessment in Individual Studies

Two reviewers independently assessed the risk of bias in the included studies using the RoB‑2 tool for RCTs [23] and the ROBINS-I tool for non-randomized trials [24]. Their assessments were then compared, and any disagreements were resolved through discussion and consultation with the third author (ASB) until consensus was achieved. For RCTs, bias was evaluated across the following domains and categorized as “low,” “high,” or “some concerns”: randomization process, deviations from intended interventions, missing outcome data, outcome measurement, and selection of reported results. The overall risk of bias of the included studies was evaluated as follows: "low risk of bias" if all fields were at low risk of bias; "some concerns" if at least one domain was evaluated as "some concern" but not at high risk of bias for any domain; "high risk of bias" if at least one or more fields were evaluated at high risk of bias, or if there were some concerns for multiple domains, in a way that notably undermines the reliability of the results.

For non-randomized trials, the following domains were evaluated: bias arising from confounding, participant recruitment methods, categorization of interventions, deviations from intended interventions, missing data, the measurement of outcomes, and bias arising from the selection of the reported outcomes. Overall risk-of-bias assessment of non-randomized included studies was evaluated as follows: low if all domains were low risk; moderate if domains were consistently low or moderate; serious if at least one domain was serious but none critical; critical if any domain was critical; and no information if the study lacked serious or critical ratings but had incomplete data in key domains.

The strength of the evidence was assessed using the GRADE (Grading of Recommendations Assessment, Development and Evaluation) framework, which classified the certainty of outcomes as high, moderate, low, or very low [25].

Data Synthesis

Review Manager (RevMan), Version 5.4 (The Cochrane Collaboration, The Nordic Cochrane Center, Copenhagen, Denmark), was utilized to perform the meta-analysis. Continuous outcomes were analyzed with a random‑effects model, based on the inverse variance method, reporting mean differences and 95% confidence intervals. Heterogeneity was considered significant at p < 0.05, and the I² index was used to quantify its magnitude. Forest plots were generated to present findings graphically. Evidence quality was systematically evaluated using the GRADE approach.

Results

Study Selection

**Figure 1:**
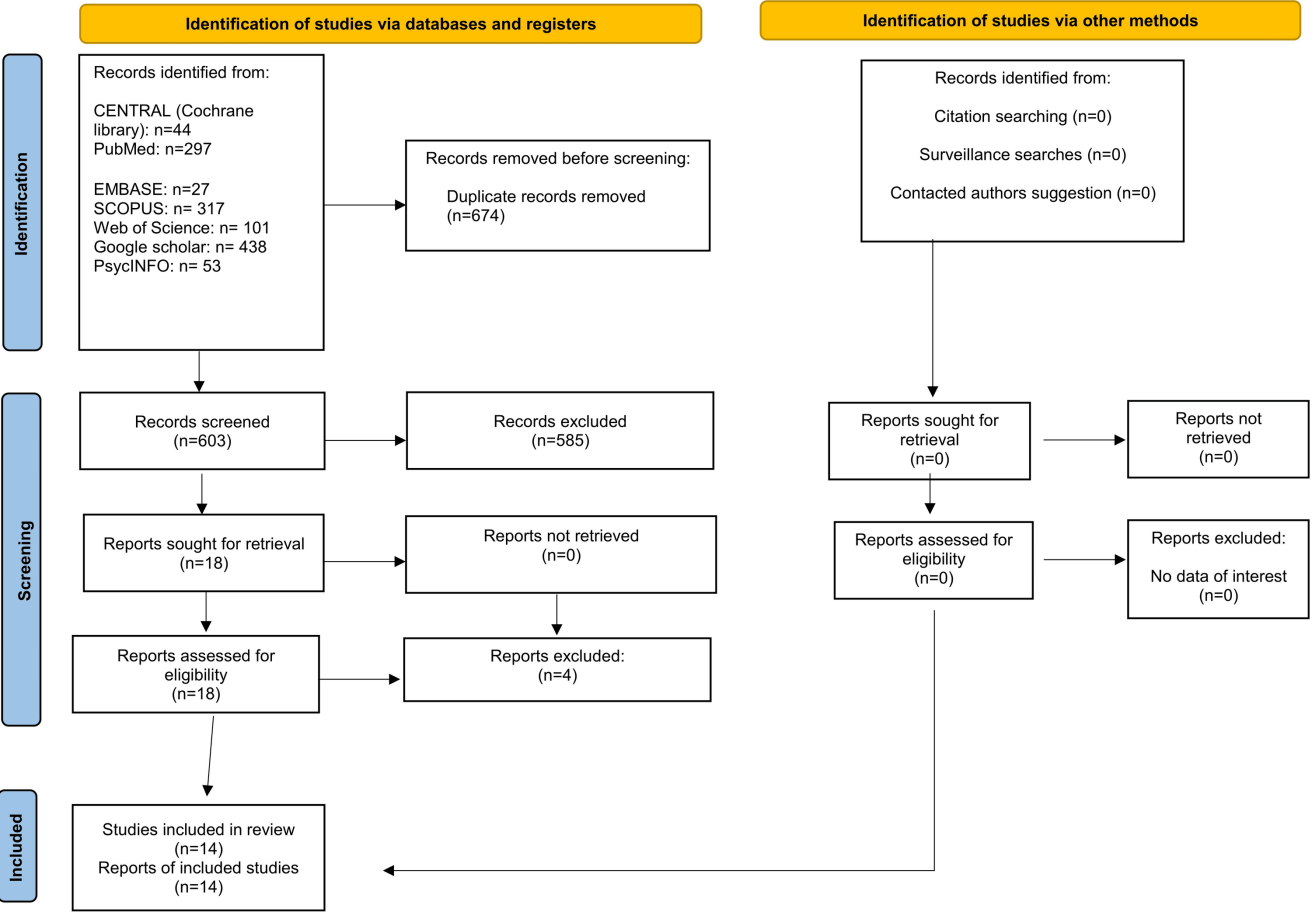
Preferred Reporting Items for Systematic Reviews and Meta-Analyses (PRISMA) flow diagram illustrating the selection of included studies

After conducting the electronic and manual searches, 1,275 articles were identified; the number dropped to 603 after duplicates were removed. After reviewing the titles and abstracts of these publications, 18 potential articles remained, and those that did not meet the inclusion criteria were excluded. Upon reviewing the full text of all 18 studies, four were found not to meet the inclusion criteria. The reasons for excluding each study are explained in Appendix 2. Following this selection process, 14 papers were included in the qualitative synthesis of the data. Figure 1 shows the PRISMA flow diagram of the reviewing process. 

Characteristics of the Included Studies

The characteristics of the included studies are summarized in Table 2. Fourteen studies were included in this review, comprising seven RCTs [7,9,12,26-29], three CCTs [30-32], three cohort studies [33-35], and one cross-sectional study [36]. A total of 1,022 patients were included across all studies, among whom 396 received treatment with conventional fixed orthodontic appliances, while the remainder received other orthodontic modalities (such as clear aligners or lingual appliances). Across all included studies, both males and females were represented - with females comprising the majority in most samples (61.25%) - and no study was conducted on a single-gender population. The male-to-female ratio was approximately 2:3. The mean age of participants across the included studies ranged from approximately 18.87 to 38 years, with most samples concentrated in the 20s to early 30s.

**Table 2:** Characteristics of the included studies M: male; f: female; RCT: randomized controlled trial; CCT: controlled clinical trial; FAT: fixed appliance treatment; CAT: clear aligners; LFA: lingual fixed appliance; IN: Invisalign®; LFBs: low-friction brackets; SLBs: self-ligating brackets; LFBO: low-friction brackets + Orthospeed® gel; LLT: low laser therapy; CORT: corticision; MAA: modified aligner appliance; VAS: visual analog scale; OHRQoL: oral health-related quality of life; GOHAI: the Geriatric Oral Health Assessment Index

Authors	Study design	Number of patients/mean age	Intervention	Outcomes	Follow-up
Miller et al. (2007) [35]	Prospective cohort	60 patients FAT: 27 (6m, 21f) 28.6 ± 8.7, CAT: 33 (11m, 22f) 38 ± 12.4	Fixed appliance treatment vs clear aligner therapy	Oral health-related quality of life (OHRQoL) (GOHAI)	Every day in the first week of treatment
Wu et al. (2010) [32]	CCT	60 patients: FAT: 30 (12m, 18f) 20.33 ± 4.205, LFA: 30 (8m, 22f) 21.63 ± 2.23	Fixed appliance treatment vs lingual fixed appliance	Pain assessed via VAS	1 week after appliance placement, 1 month, 3 months
Johal et al. (2018) [34]	Prospective cohort	58 patients (26m, 32f), 34.7 ± 12.1	Fixed appliance treatment	Pain assessed via VAS	4 h, 24 h, 3 days, and 7 days after each of 3 appointments (T0: bond-up, T1: first adjustment, T2: second adjustment)
Antonio-Zancajo et al. (2020) [31]	CCT	120 patients: FAT: 30 (13m, 17f), 24.7 ± 4.1; LFB: 30 (12m, 18f), 28 ± 9.7; LFA: 30 (13m, 17f), 33.8 ± 8.2; CAT: 30 (16m, 14f), 33.4 ± 5.1	Fixed appliance treatment vs low-friction brackets vs lingual fixed appliance vs clear aligner therapy	1. Pain assessed via VAS, 2. Oral health-related quality of life (OHRQoL) (OHIP-14)	Pain assessment: 4 h (T4h), 8 h (T8h), 24 h (T1), 2 days (T2), 3 days (T3), 4 days (T4), 5 days (T5), 6 days (T6), 7 days (T7) OHRQoL assessment: after one month of treatment
Diddige et al. (2020) [28]	RCT	36 patients (18-30 years): FAT: 12 (6m, 6f), SLBs: 12 (6m, 6f), CAT: 12 (6m, 6f)	Fixed appliance treatment vs self-ligating Damon brackets vs clear aligner therapy	Pain assessed via VAS	4 hours (T1), 24 hours (T2), Day 3 (T4), and Day 7 (T5)
Curto et al. (2020) [27]	RCT	90 patients: FAT: 30 (9m, 21f) 23.5 ± 12.6, LFBs: 30 (13m, 17f) 18.87 ± 3.6, LFBO: 30 (13m, 17f) 22.6 ± 9.5	Fixed appliance treatment vs low-friction brackets vs low-friction brackets + Orthospeed® gel	1. Pain assessed via VAS, 2. Oral health-related quality of life (OHRQoL) (OHIP-14)	Pain assessment: 4 hours (T1), 8 hours (T2), 24 hours (T3), 2 days (T4), 3 days (T5), 4 days (T6), 5 days (T7), 6 days (T8), and 7 days (T9) after the start of the treatment. OHRQoL assessment: after one month of treatment
Alcón et al. (2021) [30]	CCT	140 patients: FAT: 70 (35m, 25f) 26.97 ± 7.23, CAT: 70 (33m, 37f) 31.74 ± 11.39	Fixed appliance treatment vs clear aligner therapy	Pain assessed via VAS	Every month after the patient's monthly follow-up visit at 4 h (T1), 8 h (T2), 24 h (T3), 2 days (T4), 3 days (T5), 4 days (T6), 5 days (T7), 6 days (T8), and 7 days (T9), during the twelve-month study
Gao et al. (2021) [33]	Prospective cohort	110 patients: FAT: 55 (13m, 42f) 24.6 ± 5.2, CAT: 55 (13m, 42f) 26.0 ± 5.47	Fixed appliance treatment vs clear aligner therapy	1. Pain assessed via VAS, 2. Oral health-related quality of life (OHRQoL) (OHIP-14)	Pain assessment: every day from day 1 to day 14, OHRQoL assessment: day 1, day 7, day 14
Curto et al. (2022) [36]	Cross-sectional	120 patients (52m, 68f), 19-45 years	Conventional fixed appliance	Oral health-related quality of life (OHRQoL) (OHIP-14)	One month after the start of the treatment
Jaber et al. (2022) [12]	RCT	36 patients: FAT: 18 (8m, 10f) 20.86 ± 1.98, CAT: 18 (9m, 9f) 21.27 ± 1.87	Fixed appliance treatment vs clear aligner therapy	Oral health-related quality of life (OHRQoL) (OHIP-14)	Before treatment (T0), 1 week (T1), two weeks (T2), one month (T3), six months (T4), and 12 months (T5) after starting the treatment
Sirri et al. (2022) [9]	RCT	52 patients: FAT: 26 (6m, 20f) 21.30 ± 1.49, CORT: 26 (8m, 18f) 21.46 ± 1.76	Fixed appliance treatment vs Fixed appliance treatment with corticision	Pain assessed via VAS	After one day of the onset of treatment (T0), seven days (T1), and 14 days (T2)
Tunca et al. (2024) [29]	RCT	60 patients: FAT: 30 (15m, 15f) 21.3 ± 3.37, CAT: 30 (15m, 15f) 23.65 ± 6.58	Fixed appliance treatment vs clear aligner therapy	1. Pain assessed via VAS, 2. Oral health-related quality of life (OHRQoL) (OHIP-14)	Pain assessment: at 0, 2, and 6 h after treatment and on days 1, 3, 7, 14, and 21. OHRQoL assessment: after the start of treatment (T1) and after 10 days (T10), and 20 days (T20) of treatment
Alhafi et al. (2024) [7]	RCT	36 patients: FAT: 18 (6m, 12f) 20.94 ± 2.38, MAA: 18 (5m, 13f) 21.89 ± 2.63	Fixed appliance treatment vs modified aligner appliance	Oral health-related quality of life (OHRQoL) (OHIP-14)	Before beginning the treatment (T0), two weeks (T1), one month (T2), two months (T3) after the beginning of the treatment, and at the end of the treatment (T4)
Alfawal et al. (2024) [26]	RCT	44 patients: FAT: 22 (3m, 19 f) 24.22 + 2.99, CAT: 22 (5m, 17f) 25.40 ± 2.87	Fixed appliance treatment vs clear aligner therapy	Oral health-related quality of life (OHRQoL) (OHIP-14)	Before the start of treatment (T0), 1 week (T1), 1 month (T2), 3 months (T3), 6 months (T4), after the initial bonding of the fixed appliance or delivery of the first aligner, and post-treatment (T5)

Fixed appliance treatment (FAT) was the primary treatment modality in all included studies. Several studies incorporated comparative groups, including clear aligner therapy (CAT) [12,26,28-31,33,35], LFAs [31,32], low-friction brackets (LFBs) [31], and SLBs [28]. Additionally, corticision as an adjunctive acceleratory therapy [9] and hybrid approaches- such as the integration of Orthospeed® gel [27] were also explored; however, two studies assessed the effects of FAT independently [34,36].

Regarding pain assessment, 9 of the 14 included studies measured pain using the VAS [9,27-34]. Seven of the nine studies initiated pain assessment within the first few hours following appliance placement [9,27-31,34], one study began on the following day [33], and one study commenced evaluation one week after appliance placement [32]. Patient follow-up durations varied across the nine studies, ranging from 7 days [27,28,31] to 12 months [30]. Most studies followed patients for short periods (e.g., one to five weeks) [9,27-29,31,33], while others extended follow-up to 3 [32] or 12 months [30]. However, one study reported follow-up based on three appointments [34].

Regarding OHRQoL assessment, 9 of the 14 included studies evaluated OHRQoL using validated instruments at different stages of orthodontic care [7,12,26,27,29,31,33,35,36]. Three out of the nine studies conducted the initial assessment before starting treatment [7,12,26], while three studies conducted the assessment one month after treatment began, and this was the only assessment [27,31,36]. Three studies conducted the initial assessment on the first day of treatment [9,33,35]. Follow-up durations for OHRQoL varied across studies, ranging from 1 week [35] to 12 months [12], and, in some cases, extended until treatment completion [7,26].

Notably, pain and OHRQoL assessments were included in 4 of the 14 studies to provide a more comprehensive evaluation of patient-centered outcomes [27,29,31,33].

Risk of Bias Within Studies

**Figure 2:**
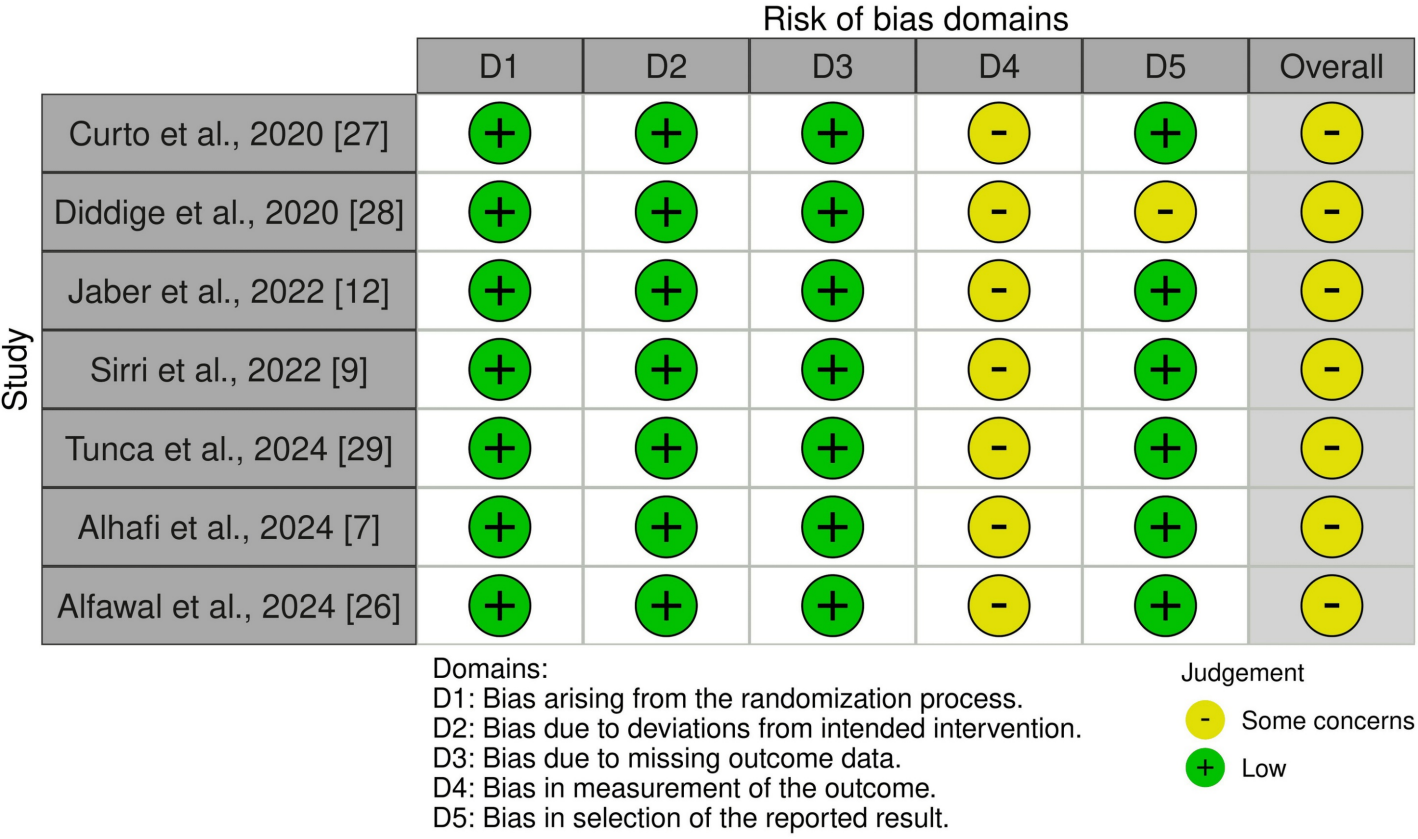
Risk of bias of the included randomized controlled trials

**Figure 3:**
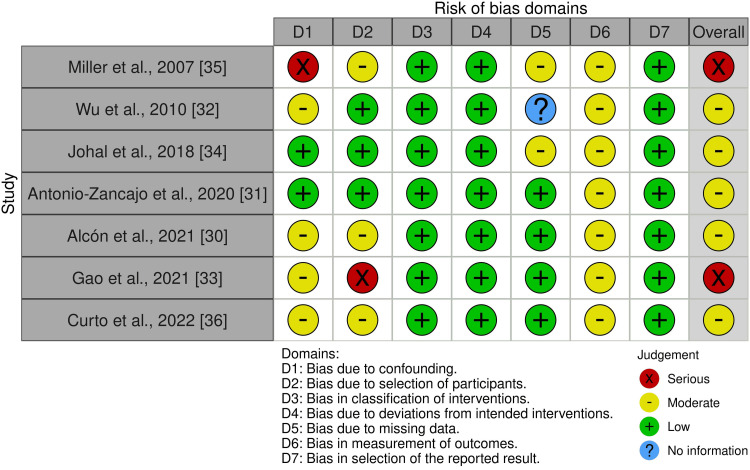
Risk of bias of the included non-randomized controlled trials

Risk of bias was assessed for seven RCTs using the ROB 2 tool. All were rated as having “some concerns,” most commonly driven by lack of participant blinding and outcome-measurement domain concerns for self-reported pain and OHRQoL [7,9,12,26-29]. For the seven non-randomized studies, the ROBINS-I tool was applied: five were judged to have a moderate risk of bias [30-32,34,36], while two were considered at serious risk [33,35]. The “serious” ratings were principally due to selection bias/confounding from non-random allocation or self-selection of treatment (e.g., socioeconomic differences between aligner and bracket groups) and baseline imbalances not adjusted for in analyses. Figures 2-3 illustrate the overall risk of bias assessments, with the rationale for each judgment detailed in Appendix 3.

Effects of Interventions

Table 3 presents a summary of the main findings. A key consideration when interpreting results is the variation in methodological approaches across studies. The included studies employed different summary metrics; the majority presented means [9,12,26-33,35,36] (suitable for normally distributed data) while two studies reported medians (more robust for skewed distributions, potentially reflecting non-normal data or an intent to assess peak pain intensity and the peak impact on quality of life) [7,29]. This methodological difference should be considered when interpreting the results.

**Table 3:** Main findings of the included trials D: day; W: week; M: month; GOHAI: the Geriatric Oral Health Assessment Index; OHIP-14: Oral Health Impact Profile - 14 Values are mean ± standard deviation unless otherwise indicated. * denotes standard deviation not reported in the original study; † denotes median.

Authors	Pain (VAS 0-100 mm)					Oral health-related quality of life (OHRQoL)					
	1D	1W	2W	1M	3M	Assessment tool	1D	1W	2W	1M	3M
Miller et al. (2007) [35]	-	-	-	-	-	GOHAI	38 ± 23.38	59 ± 10.39	-	-	-
Wu et al. (2010) [32]	-	59.2 ± 11	-	32.3 ± 8	9.7 ± 3.5	-	-	-	-	-	-
Johal et al. (2018) [34]	26.35 ± 25.92	16.02 ± 21.75	-	-	-	-	-	-	-	-	-
Antonio-Zancajo et al. (2020) [31]	50 ± 27	13 ± 16	-	-	-	OHIP-14	-	-	-	3.8 ± 2.1	-
Diddige et al. (2020) [28]	55.3*	24.9*	-	-	-	-	-	-	-	-	-
Curto et al. (2020) [27]	45 ± 22	18 ± 19	-	-	-	OHIP-14	-	-	-	3.0 ± 1.9	-
Alcón et al. (2021) [30]	43.66 ± 19.47	16.49 ± 13.87	-	21.75 ± 1.984	6.42 ± 10.09	-	-	-	-	-	-
Gao et al. (2021) [33]	42 ± 22.25	17 ± 13.3	9 ± 10.4	-	-	OHIP-14	16 ± 12.6	11 ± 10.4	8.5 ± 8.9	-	-
Curto et al. (2022) [36]	-	-	-	-	-	-	-	-	-	11.93 ± 2.19	-
Jaber et al. (2022) [12]	-	-	-	-	-	OHIP-14	-	22.88 ± 9.60	16.41 ± 9.27	14.12 ± 9.07	-
Sirri et al. (2022) [9]	19.54 ± 7.76	2.231 ± 2.61	0.07 ± 0.27	-	-	-	-	-	-	-	-
Tunca et al. (2024) [29]	56.7†	23.7†	13.3†	-	-	OHIP-14	18.73 ± 7.75	-	14.53 ± 7.07	-	-
Alhafi et al. (2024) [7]	-	-	-	-	-	OHIP-14	-	19.94†	-	16.22†	-
Alfawal et al. (2024) [26]	-	-	-	-	-	OHIP-14	-	25.18 ± 4.15	-	15.59 ± 2.91	11.46 ± 2.63

Pain evaluation: Regarding pain assessment, 9 of the 14 studies evaluated pain associated with fixed orthodontic treatment using the VAS [9,27-34]. The highest pain levels were consistently observed at the 24-hour mark. High values were reported across multiple studies, including 55.3 [28] and 50 ± 27 [31]. Tunca et al. reported a median pain score of 56.7 [29]. Other studies recorded initial mean values ranging from 26.35 ± 25.92 to 45 ± 22 [27,30,33,34]. Sirri et al. reported the lowest initial pain level, with a mean of 19.54 ± 7.76 [9].

**Figure 4:**
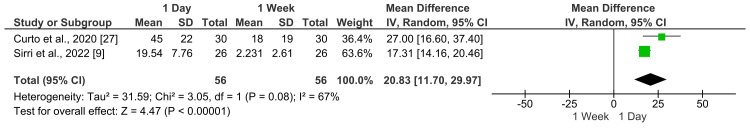
Forest plot showing the difference in pain intensity between the first day and the first week

A significant reduction in pain occurred after one week. A meta-analysis of two studies confirmed this observation (since they were the only ones reporting mean VAS scores with standard deviations at both the 24-hour and one-week time points), showing that pain was significantly higher on the first day compared to after one week (MD = 20.83; 95% CI: 11.7, 29.97; p < 0.00001; I² = 67%; Figure 4) [9,27]. It was not feasible to perform a sensitivity or subgroup analysis, since only two studies were included. This trend was supported by other studies, in which pain scores decreased to 13 ± 16 [31], 16.49 ± 13.87 [30], and 17 ± 13.3 [33]. Three studies confirmed a continued decrease at the two-week mark, with values of 13.3 [29], 9 ± 10.4 [33], and a notably low value of 0.07 ± 0.27 in Sirri et al.'s study [9]. Long-term assessment showed variable patterns. Wu et al. reported mean pain values of 32.3 ± 8 at one month, decreasing to 9.7 ± 3.5 by three months [32]. In Alcón et al.'s study, they did not measure pain at a single endpoint for each month; instead, they calculated an overall mean from all measurements taken throughout the first month (21.75 ± 1.98), and similarly for the third month (6.42 ± 10.09) [30].

OHRQoLevaluation: Regarding assessment of oral health-related quality of life (OHRQoL), 9 of the 14 studies evaluated OHRQoL [7,12,26,27,29,31,33,35,36]. In eight of the nine studies, the Oral Health Impact Profile-14 (OHIP-14) was used [7,12,26,27,29,31,33,36], while one study used the Geriatric Oral Health Assessment Index (GOHAI) [35]. The assessment of OHRQoL across the included studies showed a consistent trend of initial impairment, followed by significant improvement over time.

The timing of the initial assessment influenced the baseline scores. Two studies measuring OHRQoL after one day of treatment reported scores of 16.0 ± 12.6 and 18.73 ± 7.75 [29,33]. In contrast, studies that conducted their first post-treatment evaluation at the one-week mark reported notably higher scores, indicating greater initial impairment, with values of 22.88 ± 9.60 [12] and 25.18 ± 4.15 [26]. It is important to note that these studies [12,26] established a pre-treatment baseline (T0) before reporting these one-week values. Another study also established a pre-treatment baseline, with its first post-treatment assessment conducted at the two-week mark, reporting a median score of 19.94 [7]. By the two-week point, three studies demonstrated substantial improvement, with scores ranging from 8.5 ± 8.9 to 16.41 ± 9.27 [12,29,33].

**Figure 5:**
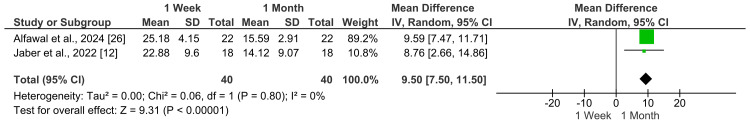
Forest plot showing the difference in oral health quality of life between the first week and the first month

The conducted meta-analysis of two studies confirmed this pattern of recovery (as they were the only ones that provided mean OHIP-14 scores with standard deviations at both the one-week and one-month assessments), showing that OHRQoL was significantly better at one month than at one week. The pooled estimate showed that quality of life after one month was significantly better by 9.50 compared to one week, and the heterogeneity was low (MD = 9.50; 95% CI: 7.50, 11.50; p ˂ 0.00001; X² = 0.06; p = 0.80; I² = 0%; Figure 5) [12,26]. Given the zero heterogeneity, there was no need for further analyses. Data from four of the nine studies at the one-month mark revealed a notable divergence in scores. Curto et al.'s study reported a mean of 11.93 ± 2.19 [36], Alhafi et al. reported a median of 16.22 [7], while the other two reported markedly lower scores of 3.8 ± 2.1 [31] and 3.0 ± 1.9 [27]. Alfawal et al. reported a three-month OHRQoL score of 11.46 ± 2.63 [26]. The study by Miller et al., which used the GOHAI scale, confirms the same trend, showing an improvement in quality of life during the first week of treatment (59 ± 10.39) compared to the first day (38 ± 23.38) (in this scale, the highest values indicate better quality of life) [35].

Certainty of the Evidence (GRADE)

The overall certainty of the evidence for the primary outcomes was assessed using the GRADE approach (Table 4). For the outcome of pain intensity, the quality of evidence was rated as very low, downgraded due to serious risk of bias, serious inconsistency (substantial heterogeneity across studies), and serious imprecision (small sample sizes and wide confidence intervals). For the outcome of OHRQoL, the quality of evidence was generally rated as low to very low. The comparison between OHRQoL at one week versus one month had a low certainty level, downgraded for risk of bias and imprecision, but not for inconsistency.

**Table 4:** Summary of the findings based on the Grading of Recommendations, Assessment, Development, and Evaluations framework ^a^ downgraded for risk of bias, ^b^ downgraded for inconsistency, ^c ^downgraded for imprecision. VAS: Visual Analog Scale; OHIP-14: Oral Health Impact Profile - 14; OHRQoL: oral health-related quality of life

Outcome time point	Quality assessment criteria						Number of patients, relative effect, and certainty			Comments
	No. of studies	Risk of bias	Inconsistency	Indirectness	Imprecision	Other considerations	No. of patients	Relative (95% CI)	Certainty	
Pain (VAS) - at 24 hours	8	Serious	Serious	Not serious	Serious	None	311	-	Very Low ⊕⊖⊖⊖^a b c^	High pain levels are consistently reported
Pain (VAS) - Day 1 vs Week 1	2 RCTs	Serious	Serious	Not serious	Serious	None	56	Relative effect (Cl 95%): MD 20.83 (11.7, 29.97)	Very Low ⊕⊖⊖⊖^a b c^	-
Pain (VAS) - 2 weeks	3	Serious	Serious	Not serious	Serious	None	111	-	Very Low ⊕⊖⊖⊖^a b c^	-
Pain (VAS) - at 1 month	3	Serious	Serious	Not serious	Serious	None	100	-	Very Low ⊕⊖⊖⊖^a b c^	Pain levels show variable patterns
Pain (VAS) - at 3 months	2	Serious	Serious	Not serious	Serious	None	100	-	Very Low ⊕⊖⊖⊖^a b c^	Low pain levels are consistently reported
OHRQoL (OHIP-14) - at 24 hours	2	Serious	Serious	Not serious	Serious	None	85	-	Very Low ⊕⊖⊖⊖^a b c^	Indicates initial impairment. Scores ranged from 16 to 18.73
OHRQoL (OHIP-14) - at 1 week	4	Serious	Serious	Not serious	Serious	None	113	-	Very Low ⊕⊖⊖⊖^a b c^	-
OHRQoL (OHIP-14) - at 2 weeks	3	Serious	Serious	Not serious	Serious	None	103	-	Very Low ⊕⊖⊖⊖^a b c^	Shows improvement from baseline. Scores range from 8.5 to 14.53
OHRQoL (OHIP-14) - Week 1 vs Month 1	2 RCTs	Serious	Not serious	Not serious	serious	None	40	Relative effect (Cl 95%): MD 9.5 (7.5, 11.5)	Low ⊕⊕⊖⊖^a c^	Meta-analysis shows significant improvement
OHRQoL (OHIP-14) - at 3 months	1 RCT	Serious	Serious	Not serious	Serious	None	22	-	Very Low ⊕⊖⊖⊖^a b c^	-

Discussion

This systematic review provides a focused analysis of pain and OHRQoL, specifically in adult patients undergoing fixed orthodontic treatment. While previous reviews have often examined mixed-age populations or compared appliance types [2,5,16,17], our findings offer a distinct perspective by concentrating on the adult demographic, whose pain perception and psychosocial responses may differ notably from those of younger patients.

The results showed a predictable adaptation pattern, characterized by an initial peak in pain at 24 hours, followed by a significant decline within the first week, and a parallel improvement in OHRQoL by the one-month mark. The improvement in OHRQoL reflects a complex interplay beyond pain adaptation. While the meta-analysis confirmed a statistical association between pain reduction and improvement in OHRQoL, the mechanisms underlying this improvement in quality of life extend beyond pain adaptation alone and were not consistently explored in the included studies. The recovery in OHRQoL may be attributed to patients' psychological adaptation to the appliance and their growing acceptance of the treatment process over time.

However, these findings should be interpreted with caution, as substantial heterogeneity was observed in the methodology of the included studies. A primary source of complexity arose from notable differences in the timing of initial outcome assessments across the studies. Notably, the timing of the initial outcome assessment varied considerably between and within the pain and OHRQoL measures. For pain, the first evaluation was conducted at different points after appliance placement: within the first few hours [27-29,31,34], at 24 hours [9,33], or after one week [32]. In contrast, the initial assessment of OHRQoL was even more heterogeneous. While some studies reported the first absolute values after one day [29,33] or one week [35], others established a pre-treatment baseline (T0), making their first post-treatment scores at one week [12,26] or two weeks [7] measures of change.

This fundamental discrepancy in baseline definition and assessment timing complicates the comparison of absolute scores across studies and likely contributed to the observed heterogeneity in reported values. Furthermore, a notable divergence in reported pain levels was observed even at similar time points. It can be attributed not only to assessment timing but also to other clinical and methodological factors. For example, the study by Sirri et al. reported notably lower pain scores across all evaluation periods compared to other studies, which could be influenced by specific patient demographics, the type of adjunctive procedure used (corticision), or inherent differences in pain perception thresholds within the sample [9]. This inter-study variability highlights that, while the overall pattern of pain resolution is consistent, pain magnitude is context-dependent and should not be generalized without considering these modifying factors.

Another point to discuss is the variation in summary metrics across the included studies. The majority presented means and standard deviations, suitable for normally distributed data. However, some studies reported medians [7,29], a practice often indicative of skewed data distributions, which is common for subjective outcomes like pain intensity that may not follow a normal curve. The use of medians in such cases is more robust for representing central tendency, but it poses a challenge for meta-analysis, which typically relies on means and standard deviations to pool data. This inconsistency limited the number of studies that could be included in our quantitative synthesis. The combination of these factors - variable assessment timing, divergent absolute scores, and the use of different or incomplete summary statistics - undoubtedly influenced the overall results of the review, leading to observed heterogeneity and limiting the precision of the pooled estimates.

In terms of the clinical significance of the findings, the statistically significant reduction in pain (a mean difference of over 20 VAS points from day 1 to week 1) and the improvement in OHRQoL (a mean difference of 9.5 OHIP-14 points out of the overall 54 points) are substantial enough to be considered clinically significant, representing a clear transition from high discomfort to a more acceptable state. However, the overall confidence in these findings is tempered by the methodological limitations discussed, which are reflected in the “low” to “very low” GRADE ratings. The risk of bias, particularly concerning self-reported outcomes in often unblinded studies, further underscores the need for cautious interpretation.

Limitations of the current work

This review has several limitations to consider, primarily the high risk of bias in several studies. The limited number of studies included in the meta-analysis was due to variability in assessment timing and statistical reporting. The reliance on self-reported outcomes, combined with the inability to blind participants, raises concerns regarding measurement bias. Furthermore, the inconsistent use of summary metrics limited the robustness and generalizability of the findings. These limitations, alongside generally small sample sizes and unaccounted-for demographic variables, underscore the need for cautious interpretation of the results.

## Conclusions

Based on very low- to low-certainty evidence, this review suggests a consistent pattern of pain peaking at 24 hours and subsiding within the first week, alongside the parallel improvements in quality of life in adult patients undergoing conventional fixed orthodontic therapy. Clinicians may use these findings cautiously to reassure adult patients that initial discomfort is normal and temporary. Recommendations should be tempered due to the limited strength of the supporting evidence.

The very low- to low-certainty evidence raises the need for more studies. Future research should prioritize standardized methodology, including consistent timing of outcome assessments and reporting of summary data, to enable more reliable meta-analyses. Investigating long-term outcomes and the impact of specific demographic and clinical factors through well-designed longitudinal studies is essential to generate higher-certainty evidence.

## Appendices

Appendix 1

**Table 5:** Electronic search strategy in this systematic review

Database	Search strategy
PubMed®	#1 (fixed orthodontic appliance OR fixed appliance OR conventional braces OR metallic braces OR conventional fixed appliance OR self-ligating braces OR lingual appliance OR clear aligners OR Invisalign) #2 (levelling OR alignment OR extraction OR retraction OR canine retraction OR anterior segment retraction OR intrusion OR extrusion OR expansion OR distalization) #3 (pain OR swelling OR tension OR pressure OR soreness OR pain perception OR pain intensity OR pain assessment OR discomfort OR pressure sensation OR pain threshold OR pain duration OR VAS OR visual analog scale OR numerical rating scale OR NRS OR Likert OR self-reported pain OR oral health-related quality of life OR OHRQoL OR quality of life OR patient-reported outcomes OR oral health impact profile OR OHIP OR OHIP-14 OR psychosocial impact OR functional limitations OR daily life interference OR self-esteem OR patient experience) #4 (adult patients OR adults OR permanent dentition) #5 #1 AND #2 AND #3 AND #4
CENTRAL (The Cochrane Library)	#1 (fixed orthodontic appliance OR fixed appliance OR conventional braces OR metallic braces OR conventional fixed appliance OR self-ligating braces OR lingual appliance OR clear aligners OR Invisalign) #2 (levelling OR alignment OR extraction OR retraction OR canine retraction OR anterior segment retraction OR intrusion OR extrusion OR expansion OR distalization) #3 (pain OR discomfort OR soreness OR quality of life OR oral health-related quality of life OR OHQoL OR OHIP OR OHIP-14 OR patient-reported outcomes OR psychosocial impact OR functional limitations OR daily life interference) #4 (adult patients OR adults OR permanent dentition) #5 #1 AND #2 AND #3 AND #4
Web of Science™	#1 TS= (fixed orthodontic appliance OR fixed appliance OR conventional braces OR metallic braces OR conventional fixed appliance OR self-ligating braces OR lingual appliance OR clear aligners OR Invisalign) #2 TS= (levelling OR alignment OR extraction OR retraction OR canine retraction OR anterior segment retraction OR intrusion OR extrusion OR expansion OR distalization) #3 TS= (pain OR discomfort OR soreness OR quality of life OR oral health-related quality of life OR OHQoL OR OHIP OR OHIP-14 OR patient-reported outcomes OR psychosocial impact OR functional limitations OR daily life interference) #4 TS= (adult patients OR adults OR permanent dentition) #5 #1 AND #2 AND #3 AND #4
Scopus®	#1 TITLE-ABS-KEY (fixed orthodontic appliance OR fixed appliance OR conventional braces OR metallic braces OR conventional fixed appliance OR self-ligating braces OR lingual appliance OR clear aligners OR Invisalign) #2 TITLE-ABS-KEY (levelling OR alignment OR extraction OR retraction OR canine retraction OR anterior segment retraction OR intrusion OR extrusion OR expansion OR distalization) #3 TITLE-ABS-KEY (pain OR discomfort OR soreness OR quality of life OR oral health-related quality of life OR OHQoL OR OHIP OR OHIP-14 OR patient-reported outcomes OR psychosocial impact OR functional limitations OR daily life interference) #4 TITLE-ABS-KEY (adult patients OR adults OR permanent dentition) #5 #1 AND #2 AND #3 AND #4
EMBASE®	#1 (fixed orthodontic appliance OR fixed appliance OR conventional braces OR metallic braces OR conventional fixed appliance OR self-ligating braces OR lingual appliance OR clear aligners OR Invisalign) #2 (levelling OR alignment OR extraction OR retraction OR canine retraction OR anterior segment retraction OR intrusion OR extrusion OR expansion OR distalization) #3 (pain OR discomfort OR soreness OR quality of life OR oral health-related quality of life OR OHQoL OR OHIP OR OHIP-14 OR patient-reported outcomes OR psychosocial impact OR functional limitations OR daily life interference) #4 (adult patients OR adults OR permanent dentition) #5 #1 AND #2 AND #3 AND #4
PsycINFO®	#1 (fixed orthodontic appliance OR fixed appliance OR conventional braces OR metallic braces OR conventional fixed appliance OR self-ligating braces OR lingual appliance OR clear aligners OR Invisalign). mp. #2 (levelling OR alignment OR extraction OR retraction OR canine retraction OR anterior segment retraction OR intrusion OR extrusion OR expansion OR distalization). mp. #3 (pain OR discomfort OR soreness OR quality of life OR oral health-related quality of life OR OHQoL OR OHIP OR OHIP-14 OR patient-reported outcomes OR psychosocial impact OR functional limitations OR daily life interference). mp. #4 (adult patients OR adults OR permanent dentition). mp. #5 #1 AND #2 AND #3 AND #4
Google™ Scholar	(fixed orthodontic appliance OR fixed appliance OR conventional braces OR metallic braces OR conventional fixed appliance OR self-ligating braces OR lingual appliance OR clear aligners OR Invisalign) AND (levelling OR alignment OR extraction OR retraction OR canine retraction OR anterior segment retraction OR intrusion OR extrusion OR expansion OR distalization) AND (pain OR discomfort OR soreness OR quality of life OR oral health-related quality of life OR OHQoL OR OHIP OR OHIP-14 OR patient-reported outcomes OR psychosocial impact OR functional limitations OR daily life interference) AND (adult patients OR adults OR permanent dentition)

Appendix 2

**Table 6:** Excluded articles and the reasons for exclusion OHRQoL: oral health-related quality of life

Authors	Title	Reasons beyond exclusion
Rakhshan and Rakhshan (2015)	Pain and discomfort perceived during the initial stage of active fixed orthodontic treatment	The study examined oral discomfort without evaluating pain or OHRQoL, and lacked clarity regarding assessment time points
Banerjee et al. (2018)	Effect of orthodontic pain on quality of life of patients undergoing orthodontic treatment	The study included patients aged 13-18 years with a mean age of 15 years
Raevanisa et al. (2024)	Orthodontic pain is related to oral health‑related quality of life in orthodontic patients	The study includes both adolescents and adults (12-30 years) without subgroup analysis, potentially confounding age-specific outcomes
Souza et al. (2024)	Impact of treatment with orthodontic aligners on the oral health-related quality of life	The study included a wide age range (11-54 years) without isolating adult data despite a mean age of 30.96

Appendix 3

**Table 7:** Reasons beyond the judges regarding the risk of bias of the included randomized controlled trials VAS: Visual Analog Scale; OHIP-14: Oral Health Impact Profile - 14; STAI: State-Trait Anxiety Inventory

Study	Randomization process	Deviations from intended interventions	Missing outcome data	Measurement of the outcome	Selection of the reported result	Overall bias
Curto et al. (2020) [27]	Low: The study used a randomization program (randomizer.org) and reported equal allocation across groups (n = 30). Baseline characteristics (age, sex, periodontal health) were comparable.	Low: No deviations from the intended intervention were detected	Low: No dropouts were reported.	Some concerns: The lack of blinding of outcome assessors may influence reporting.	Low: All pre-specified outcomes (pain at multiple time points and OHIP-14 domains) were reported. Statistical methods were clearly described and appropriate	Some concerns
Diddige et al. (2020) [28]	Low risk: The study used computer-generated random allocation with equal distribution across groups (1:1:1), and demographic balance (age, sex) was maintained. No baseline imbalances were reported.	Low: No deviations from the intended intervention were detected	Low: No dropouts were reported	Some concerns: No blinding of outcome assessors was described	Some concerns: The study reports predefined time points (4h, 24h, day 3, day 7), but it is unclear whether all questionnaire items were analyzed or selectively reported. No trial registration or protocol was cited.	Some concerns
Jaber et al. (2022) [12]	Low: Randomization was computer-generated and allocation concealed using opaque envelopes	Low: We did not detect deviations from the intended intervention arising from the trial context.	Low: No loss to follow-up; all participants completed assessments at all time points.	Some concerns: Outcome assessor was blinded, but self-reported OHIP-14 scores are inherently subjective and may be influenced by participants’ expectations	Low: All pre-specified outcomes were reported; statistical methods were appropriate.	Some concerns
Sirri et al. (2022) [9]	Low: Randomization was computer-generated with allocation concealment via opaque envelopes	Low: We did not detect deviations from the intended intervention arising from the trial context.	Low: No dropouts were reported	Some concerns: Outcome assessor was blinded, but self-reported OHIP-14 scores are inherently subjective and may be influenced by participants’ expectations	Low: Outcomes were pre-specified and reported transparently; statistical methods were appropriate	Some concerns
Tunca et al. (2024) [29]	Low: Randomization was done using sealed envelopes with a 1:1 allocation ratio. Groups were balanced in age, gender, and arch length discrepancy	Low: We did not detect deviations from the intended intervention arising from the trial context.	Low: No dropouts were reported	Some concerns: Pain and anxiety were self-reported using validated scales (VAS, STAI), but blinding of outcome assessors was not specified.	Low: All pre-specified outcomes (pain, anxiety, quality of life) were reported across multiple time points	Some concerns
Alhafi et al. (2024) [7]	Low: Randomization was computer-generated and allocation concealment was ensured using sealed envelopes. Baseline characteristics were well balanced	Low: We did not detect deviations from the intended intervention arising from the trial context	Low: No dropouts were reported	Some concerns: Outcome assessors were blinded, but the OHIP-14 and satisfaction scores are self-reported, and lack of participant blinding may have influenced responses	Low: All pre-specified outcomes were reported. Statistical methods were appropriate and transparently described.	Some concerns
Alfawal et al. (2024) [26]	Low: Randomization was performed using a random sample table and allocation concealment was ensured via opaque, sealed envelopes. Groups were comparable at baseline	Low: We did not detect deviations from the intended intervention arising from the trial context.	Low: No participants were lost to follow-up. All randomized patients were included in the final analysis.	Some concerns: Outcome assessors were blinded, but the OHIP-14 and satisfaction scores are self-reported, and lack of participant blinding may have influenced responses	Low: All pre-specified outcomes were reported. Statistical methods were appropriate and transparently described.	Some concerns

**Table 8:** Reasons beyond the judges regarding the risk of bias of the included non-randomized studies VAS: Visual Analog Scale; OHIP-14: Oral Health Impact Profile - 14; OHRQoL: oral health-related quality of life

Study	Bias due to confounding	Bias in selection of participants into the study	Bias in classification of interventions	Bias due to deviations from intended interventions	Bias due to missing data	Bias in measurement of outcomes	Bias in selection of the reported result	Overall bias
Miller et al. (2007) [35]	Serious: Baseline differences (age, income, motivation) likely affected outcomes but weren’t properly adjusted	Moderate: Patients weren’t randomized; Invisalign users were older and wealthier, indicating selection bias	Low: The intervention groups were clearly defined.	Low: No major deviations were reported. Both groups followed standard treatment protocols	Moderate: The study does not report significant dropout or missing diary entries, but detailed handling of missing data is not described	Moderate: Outcomes were self-reported. However, the use of validated instruments strengthens reliability	Low: All pre-specified outcomes were reported	Serious
Wu et al. (2010) [32]	Moderate: No control for psychological factors or pain tolerance differences	Low: Single-center recruitment; There were no differences in baseline characteristics between the groups	Low: Clear distinction between labial and lingual appliances	Low risk: Any deviations from intended intervention reflected usual practice	No information (NI): No mention of dropouts or missing VAS scores	Moderate: Self-reported pain scores; subjective and potentially influenced by expectations	Low: The study is comparable to a well-performed randomized trial in this domain	Moderate
Johal et al. (2018) [34]	Low: There are no obvious confounding factors that indicate a clear risk of bias	Low: Inclusion/exclusion criteria were clearly defined; recruitment was consecutive	Low: All participants received standardized orthodontic treatment with a calibrated protocol	Low: Protocol adherence was monitored; deviations were minimal and documented	Moderate: 16% dropout rate; authors stated no significant difference in baseline characteristics, but no imputation	Moderate: Pain measured via VAS is subjective; self-reported analgesic use may be imprecise	Low: All planned outcomes were reported; statistical methods were transparently described	Moderate
Antonio-Zancajo et al. (2020) [31]	Low: There are no obvious confounding factors that indicate a clear risk of bias	Low: Clear inclusion/exclusion criteria; sample size calculated; groups balanced by age and sex	Low: Intervention groups clearly defined	Low: No major deviations were reported	Low risk: Data were reasonably complete	Moderate: Pain and OHRQoL measured via self-reported questionnaires (VAS, OHIP-14); subjective and potentially influenced by expectations	Low: The study is comparable to a well-performed randomized trial in this domain	Moderate
Alcón et al. (2021) [30]	Moderate: Age differences between groups (BG younger than IG)	Moderate: Inclusion/exclusion criteria were clearly defined	Low: Clear distinction between bracket and aligner groups	Low: Any deviations from usual practice were unlikely to impact the outcome	Low: Data were reasonably complete	Moderate: Pain measured via VAS is subjective and potentially influenced by expectations	Low: Monthly pain scores reported consistently; statistical tests applied	Moderate
Gao et al. (2021) [33]	Moderate: Income was significantly unbalanced between two groups due to the higher cost of clear aligners, and random assignment of treatment modalities was unfeasible	Serious: Participants self-selected treatment modality based on preference and affordability	Low: Clear distinction between bracket and aligner groups	Low: No major deviations were reported	Low: Data were reasonably complete	Moderate: Pain and OHRQoL measured via self-reported questionnaires (VAS, OHIP-14); subjective and potentially influenced by expectations	Low: All pre-specified outcomes were reported	Serious
Curto et al. (2022) [36]	Moderate: Wide age range of participants included in the study	Moderate: Single-center recruitment; the wide age range of participants included in the study	Low: All participants received conventional brackets	Low: No major deviations were reported	Low: Data were reasonably complete	Moderate: Outcomes were self-reported which may introduce subjective bias	Low: All pre-specified outcomes were reported	Moderate
